# Polyphenolic Characterization of Grape Skins and Seeds of Four Italian Red Cultivars at Harvest and after Fermentative Maceration

**DOI:** 10.3390/foods8090395

**Published:** 2019-09-06

**Authors:** Massimo Guaita, Antonella Bosso

**Affiliations:** Consiglio per la Ricerca in Agricoltura e L’analisi Dell’economia Agrarian—Centro di Ricerca Viticoltura ed Enologia, via P. Micca 35, 14100 Asti, Italy

**Keywords:** polyphenols, solvent extraction, winemaking byproducts, phloroglucinolysis, ABTS

## Abstract

Agro-industry byproducts can still contain large amounts of phenolic compounds, and one of the richest sources are grape skins and seeds as grape pomace, both fermented (red winemaking) and unfermented (white winemaking). The residual polyphenolic content depends on various factors such as grape variety, vintage, and winemaking technique. In this work, four red grape varieties cultivated in northern Italy were studied: Albarossa, Barbera, Nebbiolo, and Uvalino. The work was aimed at studying the polyphenolic composition of skins and seeds from fresh grapes and from the corresponding pomace after fermentative maceration, to assess the actual importance of the varietal differences when processing winemaking byproducts for the extraction of phenolic compounds. The skin and seed extracts were prepared by solvent extraction with a 50% hydroalcoholic solution. The polyphenolic composition of all extracts was determined by spectrophotometry and high-performance liquid chromatography (HPLC); the content and the monomer composition of condensed tannins were determined by phloroglucinolysis; the antioxidant capacity was measured with the ABTS (2,2’-azinobis-(3-ethylbenzothiazoline-6- sulfonate)) method. The antioxidant capacity was higher for the seeds than for the skins, and it was positively correlated with the condensed tannins content. Significant differences in polyphenolic composition of fresh grape skins and seeds were observed between the different cultivars. In particular, Barbera and Albarossa skins were significantly distinguished from Nebbiolo and Uvalino skins for a higher content of anthocyanins and a lower content of vanillin-reactive flavans and condensed tannins; regarding seeds, Barbera and Albarossa had a lower content of vanillin-reactive flavans, proanthocyanidins, and condensed tannins than Nebbiolo and Uvalino. The winemaking process extracted the phenolic compounds to a different extent from skins and seeds, regardless of the cultivar. The differences between cultivars in the polyphenolic profile disappeared after fermentative maceration.

## 1. Introduction

Natural polyphenolic compounds have been reported to have multiple biological activities, including cardio-protective, anti-inflammatory, anti-carcinogenic, antigenotoxic, pro-apoptotic, antiviral, and antibacterial properties attributed mainly to their antioxidant and antiradical activity [1,2,3,4,5,6]. Moreover, polyphenols can limit insulin resistance and type 2 diabetes (T2D) risk [7], and act as protective agents against atherosclerosis and brain dysfunction [8]. Natural phenols have also been reported to have excellent properties as food preservatives, due to their antimicrobial and antioxidant activities [9], as nutraceuticals [10], and for many other industrial applications (natural colorants for foods, production of cosmetics, etc.).

Grapes are one of the richest sources of natural polyphenols, among which flavonoids are the most abundant and important for wine quality. Flavonoids are characterized by a 15-carbon structural backbone C_6_-C_3_-C_6_ (aryl-propyl-aryl). They are typically produced in plants as color pigments, and as a defense mechanism in response to environmental changes (exposure to ultra-violet (UV) radiations, pathogenic invasion) [11]. Grapes flavonoids include anthocyanins, flavonols, flavanones, flavones, isoflavones, and flavan-3-ols [12]. The flavan-3-ols exist as monomers ((+)-catechin, (+)-gallocatechin, (−)-epicatechin, (−)-epigallocatechin and (−)-epicatechin-3-*O*-gallate) and also as oligomers and polymers, called flavans, condensed tannins, or proanthocyanidins, the most abundant class of soluble polyphenols in grape berries. Grape proanthocyanidins are composed by four different flavan-3-ols monomer subunits linked via 4–6 and 4–8 interflavan bonds: (+)-catechin, (−)-epicatechin, (−)-epicatechin-3-*O*-gallate and (−)-epigallocatechin, the latter only in the skins. Proanthocyanidins structures vary in monomeric composition, linkage position and size (degree of polymerization), ranging from dimers to polymers with more than 40 units [13,14,15]: based on the number of flavanic units, procyanidins are divided in oligomeric (from 3 to 10 units) and condensed procyanidins (more than 10 units) that have a molecular weight higher than 3000 [12]. The condensed tannins are present in all solid parts of grape clusters (skins, seeds, stalks), with the percentage monomer composition varying with the cultivar and other geographic, agronomic and climatic factors [15,16,17].

Agro-industry byproducts can still contain a large number of phenolic compounds, and one of the richest sources are grape skins and seeds as grape pomace after winemaking, both fermented (red winemaking) and unfermented (white winemaking) [18]. Generally, grape pomace accounts for 20–30% of the initial weight of the grape [19], and its residual content of polyphenolic compounds depends on various factors such as grape variety, vintage and winemaking technique [20,21,22]. 

The polyphenolic varietal characterization of grapes has been and still is the subject of numerous works [21,23,24], aimed mainly to oenological purposes. The winemaking process, in particular the maceration operations, can be planned in order to define “tailor-made” winemaking techniques (varietal enology): the agronomic and enological practices may be more effectively directed toward the expression of some polyphenolic classes rather than others, depending on the expected results [25,26]. Similarly, the knowledge of the polyphenolic profile of the grape pomace is at the basis of its industrial exploitation (pharmaceutical, biomedical, nutraceutical applications). In both cases, the chemical characterization of the polyphenolic fraction is preceded by its extraction from the solid parts of the berry. To date no standard extraction method has been defined, and different approaches were chosen by different authors. Part of the existing literature regarding the sample treatment is reviewed in Table 1.

Solvent extraction is one of the most frequently used techniques for the isolation of phenolic compounds [18,42,43]. Solvents such as methanol, ethanol, acetone, diethyl ether, ethyl acetate, and their combinations have been widely used for the extraction of phenolics, often as aqueous mixtures with different proportions of water [28,44,45]. Generally, ethanol (a dietary alcohol) is preferable than methanol in view of a food application of the extracts [38].

Despite the high number of recent publications on the various properties of the winemaking byproducts, the studies on the differences in polyphenolic composition between the fresh grape and the corresponding pomace due to the fermentative maceration are still scarce. Some interesting works [21,22] concerned the polyphenolic characterization of some red grape cultivars mainly cultivated in France, and of their respective pomaces remaining after vinification. In the course of the present work four red grape varieties cultivated in northern Italy have been studied: Albarossa, Barbera, Nebbiolo, and Uvalino. Barbera and Nebbiolo are the two most widely cultivated varieties in Piedmont; Uvalino is a minor variety from Piedmont that has been particularly studied for its high content in polyphenolic compounds [46,47]; Albarossa, obtained from the crossbreed between Barbera and Chatus (or Nebbiolo di Dronero) [48,49] is appreciated for its richness in anthocyanins and the intense color of wines, and its use has been spreading in recent years mainly due to its attitude to late harvests.

The polyphenolic extracts from skins and seeds of the four red grape varieties sampled from different commercial wineries were prepared starting either from the fresh grapes and from the corresponding fermented grape pomace. The work was also aimed at studying the variations in the polyphenolic composition of the grapes caused by the fermentative maceration, in order to assess whether the varietal differences between cultivars observed at harvest remained after the fermentative maceration or, rather, disappeared, thus losing any importance when processing the winemaking byproducts for the extraction of polyphenolic compounds. 

## 2. Materials and Methods 

### 2.1. Reagents

The solvents used were commercial ethanol 95% v/v for the preparation of the extracts, methanol HPLC grade (VWR Chemicals, Radnor, PA, USA) and acetonitrile HPLC grade (Merck KGaA, Darmstadt, Germany) for the high-performance liquid chromatography (HPLC) analyses. Other reagents were phloroglucinol, L-ascorbic acid, sodium acetate, Folin-Ciocalteu reagent, vanillin, hydrochloric acid 37%, formic acid 98%, phosphoric acid 85% (Sigma Aldrich Co., St. Louis, MO, USA), sodium carbonate, acetic acid 100% (Merck KGaA, Darmstadt, Germany). The standards used for the determination of the response factors for the HPLC analysis (phloroglucinolysis method) were (+)-catechin, (−)-epicatechin, (−)-epicatechin-3-*O*-gallate (Sigma Aldrich Co., St. Louis, MO, USA). C-18 solid phase extraction cartridges (Sep-Pak tC18 1g, Waters, Milford, MA, USA) were used. Ultrapure water from a Milli-Q gradient A10 instrument system (Millipore Corporation, Billerica, MA, USA) was used throughout the experiment.

### 2.2. Extraction from Grape Skins and Seeds at Harvest (Unfermented)

Whole grape samples of four red grape cultivars (Albarossa, Barbera, Nebbiolo, Uvalino) widely cultivated in Piedmont (Italy) were collected at harvest from different commercial wineries. For each cultivar, 4 kg of ripe grapes were manually processed to remove stems, dry/rotten berries and other wastes, and to separate the skins from the seeds. A part of the resulting healthy berries was used to determine the average berry weight and the average skins weight (per berry) before and after drying in oven at 35 °C for 48 h (Supplementary Table S1).

The remaining material was freeze-dried in separate aliquots for the subsequent analyses. The samples were weighed in 50 mL falcons, approximately 6–7 g each, recording the weight for the calculation of the freeze-drying yield. The samples were frozen at −80 °C to ensure rapid freezing. For freeze-drying, an Edwards freeze-dryer with RV8 vacuum pump was employed (Edwards Bolton, Lostock Office Park, Bolton BL6 4SG, England). The freeze-drying procedure resulted repeatable for all the studied cultivars (Supplementary Table S1). The freeze-dried skins were in the form of powder, and the frozen seeds were ground with a coffee grinder (1 min).

The extraction of polyphenols was performed according to the method employed in a previous work [45]: 100 mg of freeze-dried skins flour or ground seeds were extracted with 6 mL of ethanol/water (1:1). The extraction protocol was as follows: 100 mg flour and 6 mL solvent were placed in a 15 mL Corex tube, vortexed for 30 s, sonicated for 20 min (50 W, 48 kHz ± 10%), then again vortexed for 30 s. The sample was centrifuged at 18 °C for 20 min at 2880× *g* (Centrifuge 5810 R Eppendorf, Hamburg, Germany), then the supernatant (5 mL) was separated from the solid residue, and vacuum dried at 35 °C with a Genevac evaporator (EZ-2, Genevac^©^, Ipswich, UK). All extractions were performed in triplicate.

### 2.3. Extraction from Fermented Pomace

The pomaces of the four cultivars were sampled at the different wineries at racking off, after soft pressing (0.5 bar). The duration of the fermentative maceration was different for all cultivars: medium-short (7 and 10 days) for Barbera and Albarossa and long (20 and 22 days) for Nebbiolo and Uvalino.

The pomace was air-dried (48 h, 35 °C), then manually processed to separate the skins from the seeds. The skin and seed flours, obtained by grinding, were extracted with a different w/v ratio (500 mg flour in 6 mL solvent) with the mixture ethanol/water (1:1), with the same extraction protocol as reported above (see 2.2). All extractions were performed in triplicate.

### 2.4. Phenolic Profile and Radical Scavenging Activity 

#### 2.4.1. Spectrophotometric Analysis 

Total and monomer anthocyanins, total flavonoids, total polyphenols, proanthocyanidins and flavans reacting with vanillin were determined by spectrophotometry [50]:

- for total anthocyanins and total flavonoids, the extract was diluted 50-fold with hydrochloric ethanol (ethanol/H_2_O/HCl; 70:30:1) and the absorbance at 540 nm (total anthocyanins) and 280 nm (total flavonoids) was measured. The results were expressed, respectively, as malvidin and (+)-catechin equivalents.
- for monomer anthocyanins, 0.5 mL wine, diluted two-fold with 0.1 N H_2_SO_4_, was loaded onto a cartridge containing 0.5 g polyvinylpyrrolidone (PVPP) (Sigma Aldrich, St. Louis, MO), previously activated with 2 mL 0.01 N H_2_SO_4_; after washing with 0.01 N H_2_SO_4_ (2 mL), the monomer anthocyanins were eluted with hydrochloric ethanol and absorbance at 540 nm was measured. The results were expressed as malvidin equivalents.
- for total polyphenols, 1 mL extract was diluted 20-fold with water, and 1 mL of diluted sample was added to 1 mL of Folin-Ciocalteu reagent, basified with 4 mL of sodium carbonate, and filled up to 20 mL. After 90 min, the absorbance at 750 nm was measured against a blank. The results were expressed as gallic acid equivalents (GAE).
- The proanthocyanidin index was determined by measuring the total anthocyanins formed by hot acid hydrolysis of the extract. A sample of 0.1 mL extract was added to 12.5 mL ethanol and 12.5 mL 37% HCl containing 300 mg/L FeSO_4_·7H_2_O. The absorbance at 532 nm was measured before and after hydrolysis (50 min in a thermostatic bth at 100 °C), and the concentration of proanthocyanidins was determined according to a calibration curve obtained with cyanidin-3-glucoside.
- For flavans reacting with vanillin, 0.5 mL extract was diluted 10-fold with methanol, and 1 mL of diluted sample was added to 3 mL vanillin (4% in methanol) and the sample was then acidified with 1.5 mL 37% HCl. After 15 min, the absorbance at 500 nm was measured against a blank (without vanillin) and the concentration of flavans reacting with vanillin was determined according to a calibration curve obtained with (+)-catechin.

#### 2.4.2. HPLC Analysis 

Hydroxycinnamyl tartaric acids (HCTA), flavonols and monomer anthocyanins were determined by HPLC [51].

- HCTA and flavonols: samples were filtered with a 0.20 μm polypropylene filter (VWR International, Milano, Italy) and injected (10 μL). The column was an ODS Hypersil RP-C18 reversed-phase HPLC column (200 × 2.1 mm I.D., 5 μm packing, Thermo Scientific, Waltham, MA, USA), at 25 °C. The flow rate was 0.25 mL/min. Phase A was H_3_PO_4_ 0.001 M, and phase B was methanol 100% (HPLC grade). The signal was monitored at 320 nm for HCTA and 360 nm for flavonols. The concentrations of HCTA and flavonols were determined with calibration curves obtained with pure standards.
- Monomer anthocyanins were determined by HPLC after concentration on a C18 cartridge. Samples were filtered with a 0.20 μm polypropylene filter (VWR International, Milano, Italy) and injected (10 μL). The column was an ODS Hypersil RP-C18 reversed-phase HPLC column (100 × 2.1 mm I.D., 5 μm packing, Thermo Scientific, Waltham, MA, USA), at 25 °C. The flow rate was 0.25 mL/min. Phase A was 10% formic acid in water, and phase B was 10% formic acid and 50% methanol in water. The signal was monitored at 520 nm. The percentage of the individual anthocyanins in the mixture was calculated from the peaks area.
- Monomer flavan-3-ols ((+)-catechin and (–)-epicatechin) were determined by HPLC using a method for seeds [52], later adapted for wines (data not published): samples were filtered with a 0.20 μm polypropylene filter (VWR International, Milano, Italy) and injected (20 μL). The column was an ODS Hypersil RP-C18 reversed-phase HPLC column (200 × 2.1 mm I.D., 5 μm packing, Thermo Scientific, Waltham, MA, USA), at 25 °C. The flow rate was 0.25 mL/min. Phase A was H_3_PO_4_ 0.001 M and phase B was acetonitrile 100% (HPLC grade). The signal was monitored at 280 nm, and the peaks were identified according to the external standard method. The concentrations of (+)-catechin and (–)-epicatechin were determined with a six-point calibration curve obtained with pure standards. Each standard was injected in triplicate to assess both the linearity and repeatability of the method.

#### 2.4.3. Radical Scavenging Activity 

The determination of the antioxidant capacity was performed only for the grape extracts with the (2,2’-azinobis-(3-ethylbenzothiazoline-6-sulfonate)) (ABTS) method as proposed by [53], which consists of a spectrophotometric assay that measures the decrease of absorbance at 734 nm of the radical cation ABTS in the presence of antioxidant molecules. The percentage decrease of absorbance is calculated: the antioxidant capacity is expressed by comparison with the measured absorbance values for known amounts of an antioxidant molecule selected as reference standard, in this case ascorbic acid.

### 2.5. Characterization of Condensed Tannins by Phloroglucinolysis

Phloroglucinolysis is the acid-catalyzed cleavage of the condensed tannins (proanthocyanidins) in the presence of excess phloroglucinol as nucleophile molecule [26]. The method consists of a sample preparation step, followed by the phloroglucinolysis reaction and the HPLC analysis of the reaction products. The operating protocol ([54], with some modifications) was as follows. The extracts obtained during the extraction trials (5 mL) were evaporated to dryness under vacuum at 35 °C. The dry residue was dissolved in 2.5 mL of 2% aqueous acetic acid (using ultrasound, when necessary) and 1.5 mL was applied on a C-18 solid phase extraction cartridge (Sep-Pak tC18 1 g, Waters, Milford, MA, USA). Sugars and organic acids were eluted with 5 mL of 2% aqueous acetic acid, the phenolic compounds were recovered with 8 mL of methanol. The eluent volume was evaporated to dryness under vacuum, re-dissolved in 400 μL of the reactive solution (250 mg phloroglucinol +50 mg ascorbic acid in 5 mL methanol/HCl 98:2) and heated for 25 min at 50 °C (water bath). The reaction was stopped with 400 μL of aqueous 200 mM sodium acetate, and the sample was filtered with a 0.45 μm polypropylene syringe filter (Whatman Inc., NJ, USA) directly into the HPLC vial. Samples were analyzed with an Agilent 1200 HPLC system (Agilent Technologies, Palo Alto, CA, USA) with a reversed-phase Atlantis T3 dC18 column (5 μm packing, 250 × 2.1 mm i.d., Waters, Milford, MA, USA) protected by a guard column of the same material (10 × 2.1 mm i.d., Waters, Milford, MA). The mobile phase was a gradient of acetonitrile/water/formic acid (80:18:2; solvent B) in water/formic acid (98:2; solvent A), at a flow rate of 0.30 mL/min at 30 °C. Proportions of solvent B were as follows: 0–5 min with 0% B; 5–20 min, 0–6.5%; 20–31 min with 6.5%; 31–35 min, 6.5–10%; 35–65 min, 10–20%; 65–70 min with 20%; 70–75 min, 20–100%; and 75–80 min, 100–0%. The injection volume for all samples was 10 μL. The monomers (terminal subunits and extension subunit-phloroglucinol adducts) were detected by a DAD detector (Agilent G1315D), identified by comparison with the retention times and quantified from peak areas at 280 nm with the Chemstation software (Agilent Technologies, Palo Alto, CA, USA) using external calibration with known concentrations of flavan-3-ol monomers from Sigma (Saint Louis, MO, USA). 

The total condensed tannins content, their mean degree of polymerization (mDP), and the percentage of each constitutive unit were determined. The molar concentration of the subunits was calculated from their molar absorptivities, as reported by [27], and expressed as molar percentage. To calculate the apparent mDP, the sum of the terminal and extension subunits (in moles) was divided by the sum of the terminal subunits (in moles). The total condensed tannins, expressed in mg per 100 mg d.w. of seed flour, were determined as the sum of the quantified subunits. For the quantification of the different subunits in units of weight, the response factors of (+)-catechin, (−)-epicatechin and (−)-epicatechin-3-*O*-gallate were calculated by direct HPLC injection of the standards. The response factors of the extension subunits were then calculated using the response factor of (+)-catechin and considering the respective values of the molar extinction coefficients [27].

### 2.6. Statistical Analysis

All data were processed with Analysis of Variance (ANOVA) and Tukey’s test. The correlation matrix between the different parameters describing the tannins and anthocyanins content of the grapes and the ABTS index was calculated, separately for skins and seeds. Furthermore, the data regarding the polyphenolic composition were subjected to the Principal Components Analysis, separately for skins and seeds, with XLSTAT 2019 (Data Analysis and Statistical Solution for Microsoft Excel. Addinsoft, Paris, France, 2019).

## 3. Results

### 3.1. Polyphenolic Characterization of Grape Skins and Seeds at Harvest 

#### 3.1.1. Spectrophotometric Analysis of the Grape Skin and Seed Extracts

##### Skins 

Considering the polyphenolic composition as referred to the dry weight (DW) of lyophilized skin flour (Table 2), the cultivars differed significantly from one another for all the considered parameters, except for the ratio between monomer and total anthocyanins and for total polyphenols (GAE).

The concentration of total and monomer anthocyanins ranked significantly in the order Nebbiolo < Uvalino < Barbera < Albarossa. With the increase of the total anthocyanins content, the wavelength corresponding to the maximum absorbance in the red region moved to higher wavelengths (purple hues). Despite the variability of the anthocyanins content, the ratio between monomer and total anthocyanins was stable, ranging between 0.63 and 0.66 (monomer anthocyanins account for 63–66% of total anthocyanins).

Three cultivars, Nebbiolo, Uvalino, and Barbera, had a similar total flavonoid content, while Albarossa had a significantly higher flavonoid concentration. The total flavonoid content is influenced, in particular, by the concentration of anthocyanins and flavans at high and medium-low molecular weight (proanthocyanidins and flavans reactive to vanillin, respectively). In the case of Nebbiolo and Uvalino the skins were richer in proanthocyanidins and flavans reactive to vanillin but had a lower anthocyanins content, while the opposite was observed for Barbera: the result was a similar total flavonoids content for these three cultivars. On the contrary, the skins of Albarossa were particularly rich in anthocyanins and had a medium-high tannins content: the total flavonoid content was therefore higher than for the other 3 cultivars. As regards the content of proanthocyanidins (P) and flavans reactive to vanillin (V), Nebbiolo was the richest, followed by Uvalino, then Albarossa and Barbera, similar to each other. The V/P ratio was significantly higher for Nebbiolo and Uvalino than for Albarossa and Barbera.

Considering these values as referred to the weight of grapes (Table 2), the differences between cultivars changed, the concentrations being influenced by the berries size, highly variable between cultivars (Supplementary Table S1). In particular, when referring the values to the weight of grapes, the four cultivars resulted distinguished from one another as regards total flavonoids and total polyphenols, the opposite as regards proanthocyanidins.

##### Seeds

Referring data to the weight of seed flour (Table 2), the concentration of total flavonoids in the grape seed extracts was 2.9–5.1 times higher than in the skin extracts, while the concentration of total polyphenols was 2–3.1 times higher than in the skin extracts. Nebbiolo and Uvalino seeds had higher contents of flavans reactive with vanillin and total polyphenols than Barbera and Albarossa seeds, similar to each other. Uvalino differed from all the other cultivars for the significantly highest content in proanthocyanidins, followed by Nebbiolo, then Barbera and Albarossa, similar to each other. Finally, Nebbiolo and Uvalino seeds had significantly higher antiradical capacity (ABTS) than Barbera and Albarossa seeds. The seed extracts, due to the higher polyphenolic content, had ABTS values higher 2.5–3.7 times than those of the skin extracts.

Unlike what observed for the skins, in the case of the seeds the differences between cultivars remained almost unvaried when considering the results as referred to the weight of grapes. Overall, Nebbiolo and Uvalino had the highest contents in total flavonoids, flavans reactive with vanillin, proanthocyanidins, and total polyphenols, while Albarossa showed intermediate values for the same parameters, and Barbera the lowest.

#### 3.1.2. HPLC Analysis of Grape Skin and Seed Extracts

##### Skins

The hydroxycinnamyltartaric acids (HCTAs) content in the grape skins was determined by HPLC (Table 3). HCTAs are the esters of hydroxycinnamic acids (caffeic, p-coumaric and ferulic acids) with tartaric acid, which is particularly abundant in the berry juice and can also be found in the skin cells. Their concentration is influenced by the cultivar. The most abundant HCTA was trans-caffeiltartaric (or trans-caftaric) acid, followed in order of importance by its cis-isomer, then by trans- and cis-p-coumaroyltartaric (or coutaric) acid. Among the studied cultivars, the highest concentrations of trans-caftaric acid were observed in Uvalino and Barbera, followed by Albarossa and, finally, Nebbiolo. The same trend was observed for trans-coutaric acid.

Among flavonols (Table 3), significant differences between cultivars were observed for myricetin, particularly abundant in Barbera skins where it reached concentrations 2–3 times higher than in the other cultivars, more similar to each other. Nebbiolo skins had the lowest concentrations of myricetin, quercetin glucuronide, and quercetin glucoside, while Albarossa skins had the lowest content of kaempferol glucoside and glucuronide.

The anthocyanins profile is characteristic of the cultivar, and many studies have been conducted on this topic. Table 3 reports the anthocyanins profile of the four studied cultivars. Barbera skins were characterized by the highest percentage of malvidin-3-G, while Nebbiolo skins were rich in di-hydroxylated anthocyanins, in particular peonidin-3-G. Uvalino skins had a high content of malvidin-3-G and peonidin-3-G and were rich in cynnamates. The most abundant anthocyanin in Albarossa skins was malvidin-3-G, followed by delphinidin-3-G and petunidin-3-G (the 3 tri-hydroxylated anthocyanins), and the percentage of cynnamate forms was as high as for Uvalino.

Working with a different extraction method (fresh grape skins extracted in ethanol/water/HCl 70/30/1) [55], obtained similar anthocyanins profiles for Barbera and Nebbiolo: delphinidin-3-G 13%, cyanidin-3-G 5%, petunidin-3-G 14%, peonidin-3-G 6%, malvidin-3-G 40%, acetates 11%, cynnamates 11% for Barbera, and delphinidin-3-G 8%, cyanidin-3-G 15%, petunidin-3-G 6%, peonidin-3-G 37%, malvidin-3-G 28%, acetates 3%, cynnamates 7% for Nebbiolo.

##### Seeds

The concentrations of flavan-3-ols, (+)-catechin and (−)-epicatechin, in the grape seed extracts are reported in Table 3. The most abundant molecule for all cultivars was (−)-epicatechin, whose concentration was significantly the highest in Barbera seeds. As regards (+)-catechin, its content in the Nebbiolo seeds was the lowest among all cultivars, and significantly different from Albarossa and Barbera.

#### 3.1.3. Phenolic Characterization by Phloroglucinolysis of the Grape Skin and Seed Extracts 

##### Skins

The composition of the condensed tannins in the grape skin extracts was determined by phloroglucinolysis. In detail, Table 4 reports the concentration of condensed tannins, their mDP and the percentage monomer composition. The monomeric units that constitute tannins can be divided in terminal and extension units: overall, in the skin extracts 7 different units can be found, among which 3 as terminal units, (+)-catechin (C), (−)-epicatechin (EC) and (−)-epicatechin-3-*O*-gallate (ECG), and 4 as extension units, (+)-catechin (C), (−)-epicatechin (EC), (−)-epicatechin-3-*O*-gallate (ECG) and (−)-epigallocatechin (EGC). The extension units were identified by different chromatographic peaks compared to the corresponding terminal units because they are bound to phloroglucinol.

Significant differences between cultivars were observed for the proportion of the different monomer units and the mDP. For all cultivars the most abundant terminal unit was C, followed by EC, and then ECG.

The percentage weight of the terminal units was lower in the skins of Nebbiolo and Uvalino that had a mean polymerization degree significantly higher (22–24 units) than Albarossa and Barbera, similar to each other (between 14 and 15 units). The most abundant extension unit was EC: about 48% in Nebbiolo and Uvalino and 58% in Albarossa and Barbera. EGC was the second extension unit in order of importance for Nebbiolo and Uvalino (respectively 28% and 26%), while for Albarossa and Barbera the second most abundant extension unit was C (respectively 20% and 23%). The proportion of ECG ranged from 2.8% in Nebbiolo to 4.4% in Albarossa, with intermediate values of about 3.9% in Barbera and Uvalino.

Assuming 100 the sum of the units based on the two isomers (C and EC), EC was more abundant in Nebbiolo and Uvalino (respectively 79% and 78%) than in Albarossa and Barbera (respectively 74% and 72%). Consequently, the opposite was observed for C (Table 4).

Significant differences were observed in the content of condensed tannins: when referred to the DW of lyophilized skin flour (Table 4), the highest concentration was found in Nebbiolo, followed by Uvalino, Albarossa, and Barbera; when referred to the weight of grapes, the ranking order changed, in particular for Nebbiolo, and it was as follows: Uvalino > Albarossa = Nebbiolo > Barbera.

##### Seeds

The composition of the condensed tannins in the grape seed extracts was determined by phloroglucinolysis. In detail, Table 4 reports the concentration of condensed tannins, their mDP and the percentage monomer composition. In the case of seeds, the monomer molecules that constitute seed-condensed tannins are 6: (−)-epigallocatechin (EGC) is absent in the seeds.

Significant differences between the cultivars were observed for all the considered parameters. The most abundant terminal units were C in Nebbiolo and Uvalino, and EC in Albarossa and Barbera, while the most abundant extension unit was EC in all cultivars. The mDP was 4–5.6 units; lower (about 4 units) in Nebbiolo and Barbera, slightly higher in Albarossa and Uvalino (respectively 5.2 and 5.6 units).

Assuming 100 the sum of the units based on the two isomers (C and EC), EC was significantly more abundant in Uvalino (87%), followed by Albarossa, Nebbiolo, and finally Barbera. On the contrary, the highest percentages of galloilated forms (ECG) were observed in Albarossa and Barbera (about 20%), followed by Nebbiolo (18%) and Uvalino (17%) (Table 4).

Significant differences between cultivars were observed in the condensed tannins content when referred to the DW of lyophilized skin flour: higher concentrations were observed in Uvalino and Nebbiolo, followed by Barbera and Albarossa. The differences in concentration were quantitatively more important and with a higher degree of statistical significance when referred to the weight of grapes: Nebbiolo seeds had the highest condensed tannins content, significantly different from Albarossa and Barbera; Uvalino followed with an intermediate value, not significantly different from Nebbiolo and Albarossa, and finally Barbera with the lowest condensed tannins content, equal to 47% of the concentration measured for Nebbiolo.

### 3.2. Polyphenolic Characterization of Fermented Pomace

#### 3.2.1. Spectrophotometric Analysis of the Skin and Seed Extracts from Fermented Pomace

##### Skins 

The analyses of the phenolic profile were repeated for the fermented pomace sampled after racking off and soft pressing (0.5 bar). As already reported in the Materials and Methods section, the duration of the fermentative maceration was not the same for all cultivars: according to the specific (varietal) winemaking protocols, the macerations of Barbera and Albarossa were shorter than those of Nebbiolo and Uvalino, which are generally used for the production of wines destined to longer aging periods.

As regards the polyphenolic composition of the pomace skins (Table 5), the cultivars were distinguished from one another with highly significant differences for all the considered parameters.

Albarossa skins had the highest total anthocyanins content, equal to about 3, 8, and 19 times the content measured for Barbera, Uvalino, and Nebbiolo skins, respectively. The same trend was observed for the monomer anthocyanins content. As already observed for the fresh grapes, with the increase of the total and monomer anthocyanins content the wavelength corresponding to the maximum absorption in the red color increased towards purplish hues.

As regards the polyphenolic compounds, the Albarossa pomace skin extracts had the significantly highest contents in total flavonoids, total polyphenols, and proanthocyanidins. Albarossa was significantly different for all parameters from Nebbiolo and Uvalino, similar to each other, and for the total flavonoids and total polyphenols content from Barbera. On the contrary, Albarossa was significantly the poorest in flavans reactive with vanillin, while Barbera was averagely the richest. The V/P ratio was therefore significantly the lowest for Albarossa. 

##### Seeds 

The polyphenolic composition of the pomace seed extracts is reported in Table 5. Unlike what observed for the fresh grapes when, on average, the seed extracts had total flavonoids contents much higher than the skin extracts, in the case of the fermented pomace the situation was the opposite: the seed extracts had lower contents in total flavonoids, proanthocyanidins, and total polyphenols than the skin extracts.

Among the pomace seed extracts, the significantly highest concentrations for these parameters were observed for Barbera, while for Albarossa, Nebbiolo, and Uvalino these values were lower and similar to each other. Moreover, the Barbera seeds had a content in flavans reactive to vanillin significantly higher than the seeds of the other cultivars, similar to each other, but also than the skins from the same Barbera pomace.

#### 3.2.2. Phenolic Characterization by Phloroglucinolysis of the Skin and Seed Extracts from Fermented Pomace 

##### Skins

The composition of the condensed tannins in the pomace skin extracts was determined by phloroglucinolysis. In detail, Table 6 reports the concentration of condensed tannins, their mDP and the percentage monomer composition. The condensed tannins content in the pomace skins was 5.3–18.8 times lower than in the respective grape skins.

Significant differences were observed between the cultivars, but with a different ranking order compared to the fresh grapes. Strong variations were noticed for mDP, which was halved in the case of Albarossa and reduced 5-10.7 times for the other cultivars. Significant differences in the proportion of the different monomer units of the condensed tannins were observed between the cultivars.

Excluding Albarossa, the most abundant terminal unit was C, followed by EC, and then ECG. The total percentage of these terminal units was the lowest in the Albarossa extracts, which had the significantly highest mDP than the other cultivars, similar to each other (Table 6). For all cultivars, the most abundant extension unit was EC. The second extension unit in order of importance was C for Barbera, Nebbiolo, and Uvalino, while for Albarossa it was ECG. EGC, unlike what observed for the grape skins, was the less important extension unit for all cultivars.

Assuming 100 the sum of the units based on the two isomers (C and EC) (Table 6), the percentage weight of C in the pomace skin extracts was higher than in the fresh skin extracts except for Albarossa: C was almost equal to EC for Nebbiolo and slightly higher than EC for Uvalino. The ranking order of the cultivars for the percentage value of C was the opposite of the order observed for mDP.

##### Seeds

The composition of the condensed tannins in the pomace seed extracts was determined by phloroglucinolysis (Table 6). As a result of the maceration process, the condensed tannins content dropped much more markedly in the seeds than in the skins. Significant differences between cultivars were observed for the condensed tannins content: Barbera pomace seeds had a significantly higher content than the other 3 cultivars, similar to one another. After the fermentative maceration, the ranking of the cultivars according to the condensed tannin content was changed, and the differences reduced.

The mDP (Table 6) was 1.5–3.2 times lower than for the fresh seeds and the differences between cultivars, although statistically significant, were of little practical importance. Highly significant differences were observed between the cultivars in the proportion of the monomer units that constitute pomace seed-condensed tannins.

The varietal differences observed for the grape seeds were modified during the fermentative maceration: for all cultivars the most important terminal unit was C, and among the extension units the most important was EC.

Assuming 100 the sum of the units based on the two isomers (C and EC) (Table 6), EC remained the isomer significantly more abundant in the pomace seeds, although with lower percentages than in the grape seeds. In particular, a drastic decrease in the proportion of EC in favor of C was observed for Uvalino, up to values almost equivalent for the two isomers.

## 4. Discussion

The present work concerned the study of the polyphenolic composition of skins and seeds of 4 Italian red grape cultivars (Albarossa, Barbera, Nebbiolo, and Uvalino), derived from both the fresh grapes and the corresponding pomace at the end of the fermentative maceration.

The comparison between the polyphenolic composition of skins and seeds from fresh grapes allowed the evaluation of their oenological potential. The data used are those referring to the weight of the grapes (Supplementary Table S1), which take into account the size of the grapes and the quantity of must in which the polyphenolic fraction is extracted.

As regards the grape skins, the total anthocyanins content for Barbera was about 2.5 times higher than for Nebbiolo, according to what reported by [56] (ratios between 1.5 and 2.6), and it ranged between the values reported by [56] and [57] and those reported by [58]. Regarding Nebbiolo, the total anthocyanins content was similar to the values reported by [56] (480–550 mg/kg), and in our previous work [59] (476–658 mg/kg). Albarossa skins had the highest total anthocyanin content among all cultivars, ranging between the values reported by [48] (1978 mg/kg) and those reported by [49] (2660 mg/kg). For Uvalino, [46] found an average anthocyanins content of 900 mg/kg, lower than what observed during the present work, but, according to our results, higher than for Nebbiolo grapes.

As regards the polyphenolic profile of the grape skins, Albarossa was the richest in total flavonoids and total polyphenols, with concentrations similar to those reported by [48]. Nebbiolo and Uvalino, on the other hand, were the richest in flavanic compounds (condensed tannins determined with phloroglucinolysis, flavans reactive with vanillin and proanthocyanidins measured by spectrophotometry). Regarding the spectrophotometric data, for Nebbiolo the values were similar to those reported by [60], while for Uvalino the concentrations of proanthocyanidins and flavans reactive with vanillin were higher than those reported by [47]. Finally, Barbera skins were the poorest in polyphenolic compounds, and the total flavonoids content was in accordance with [56].

As regards the grape seeds, Nebbiolo and Uvalino were the richest in polyphenolic compounds, followed by Albarossa and Barbera, in accordance with the data reported in the bibliography, but in our case the measured concentrations of polyphenolic compounds were averagely higher than those reported in the bibliography. In the present work, the use of a 50% hydroalcoholic solution resulted in an increase in the extraction yields of the polyphenolic fraction from the seeds, compared to a 12% ethanol solution acidified at pH 3.4 and treated with 2 g/L of potassium metabisulphite, used by the authors who studied the same cultivars [47,48,49,56,57,58,59]. During a previous work [45] we observed that the extraction yields of the polyphenolic fraction from grape seed flour doubled when the ethanol concentration in the extracting solution increased from 25% to 50%. The same trend was observed by [28] for grape skin extracts. The fact that only for the skins our results agreed with the cited works that used the SO_2_ added 12% hydroalcoholic solution probably depends on the synergistic effect of sulfur dioxide. In fact, it is known that SO_2_ has an extracting effect of polyphenols from the skin cells [12]. On the contrary, SO_2_ has no extracting effect on grape seeds.

Finally, as regards the composition in condensed tannins of skins and seeds, determined with the phloroglucinolysis method, there are no reference data in the literature about the fresh grapes of the four studied cultivars. As already described in detail in the results, the cultivar effect is evident also on the concentration and composition of condensed tannins. The cultivar effect on the variability of polyphenolic composition concerns also other plant species, such as olive leaves [61]. To compare the polyphenolic composition of fresh grape skins and seeds with that of the corresponding fermented pomace (sampled after the end of fermentative maceration), the concentrations of the different classes of compounds were referred to the DW of flour. This method of presenting the results is currently used in the studies of chemical characterization of the oenological byproducts.

Considering the values reported by [21], the total polyphenol content (GAE) of fresh grape skins, referred to the dry weight of flour, was averagely higher in the four studied cultivars than in Grenache, Carignan, and Mourvedre, but similar to Syrah. Regarding fresh grape seeds, the GAE values were averagely 2–3 times higher; Nebbiolo and Uvalino seeds were the richest in polyphenolic compounds.

The analyses of the polyphenolic profile were repeated for the fermented pomace. The fermentative maceration caused a strong reduction in the total polyphenol content (GAE) of skins and seeds. Regarding pomace skins, GAE values dropped by 80-90%. Higher GAE losses were observed for pomace seeds, in particular for Nebbiolo, Uvalino, and Albarossa. The total polyphenol contents of the seeds were much lower than those reported by [21]. 

The correlations between the variables describing the polyphenols and anthocyanins content of the extracts and the ABTS index that measures the radical scavenging activity of the extracts were studied in order to verify which compositional parameter could be associated with the radical scavenging activity of the extracts. The correlation matrices (Supplementary Tables S2 and S3) were calculated separately for skins and seeds, respectively.

The degree of multicollinearity among variables, referred to the skins, was low (Supplementary Table S2), due to the characteristics of the cultivars considered: Albarossa and Barbera are rich in anthocyanins and poor in tannins while Nebbiolo and Uvalino are poor in anthocyanins and rich in tannins. This particular condition allowed the highlighting of differences between the variables regarding the correlations with the ABTS parameter that measures the radical scavenging capacity of the extracts. The ABTS parameter was closely related to the condensed tannins content (Pearson’s *r* = 0.866), and to the content of proanthocyanidins (*r* = 0.742) and vanillin-reactive flavans (*r* = 0.748). On the contrary, no statistically significant correlations were observed between the ABTS parameter and the GAE index and the total flavonoid content, while the correlation coefficient between ABTS and total and monomer anthocyanins was negative. On the contrary, in the case of seeds the variables that describe the polyphenolic content were all highly correlated with each other and to the ABTS index. As already reported by other authors [62], the absence of significant correlations between the GAE and ABTS values of the skin extracts may depend on the fact that the Folin-Ciocalteu method is not specific for the polyphenolic component and is therefore influenced by the presence of other compounds that do not necessarily have antiradical activity. The presence or absence of correlations between GAE and ABTS can therefore depend on the nature of the matrix used, differently from the analysis of condensed tannins which is specific for the flavan fraction.

The data matrix related to the polyphenolic profile of the skin extracts was subjected to the Principal Components Analysis (PCA). This technique, based on a reduced number of variables (Principal Components) calculated from the linear combination of the original variables, allows visualization of the spatial distribution of the skin samples of the 4 cultivars before and after fermentative maceration. The Principal Components are uncorrelated with each other and are numbered according to the decreasing order of the data variability they describe. In our case, the information contained in the original data matrix was described by 3 Principal Components, associated with a higher variability than the average variability of an original variable.

**Figure 1:**
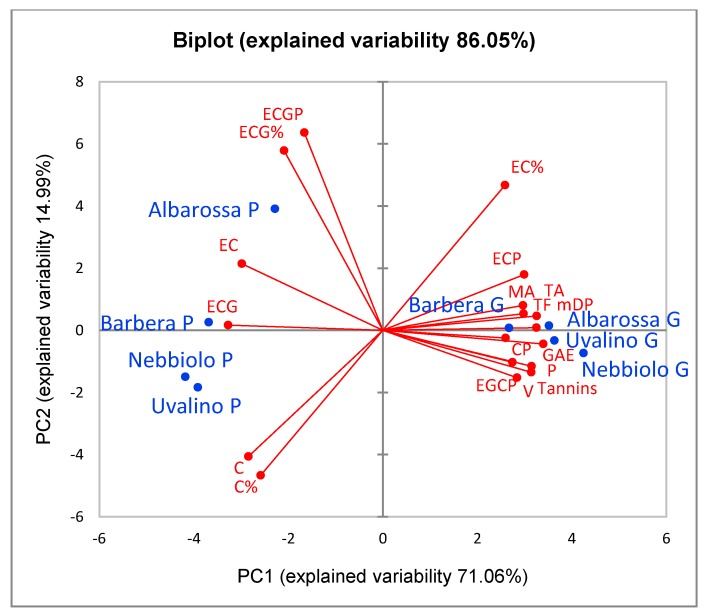
Representation of the loadings (variables) and the scores (skin extracts) in the plane defined by the 1st and 2nd Principal Components (PCA). For each cultivar (blue), the suffix G represents grape extracts, P represents pomace extracts. In red are reported the chemical variables that describe the polyphenolic profile of the extracts: GAE = total polyphenols; TF = total flavonoids; TA = total anthocyanins; MA = monomer anthocyanins; V = flavans reactive with vanillin; P = proanthocyanidins; Tannins = condensed tannins (phloroglucinolysis); mDP = mean degree of polymerization; C, EC, ECG = (+)-catechin, (−)-epicatechin and (−)-epicatechin-3-*O*-gallate present as terminal units; CP, ECP, ECGP = (+)-catechin, (−)-epicatechin and (−)-epicatechin-3-*O*-gallate present as extension units; EGCP = (−)-epigallocatechin present only as extension unit; C%, EC%, ECG% = total percentages of the monomers (terminal + extension).

**Figure 2:**
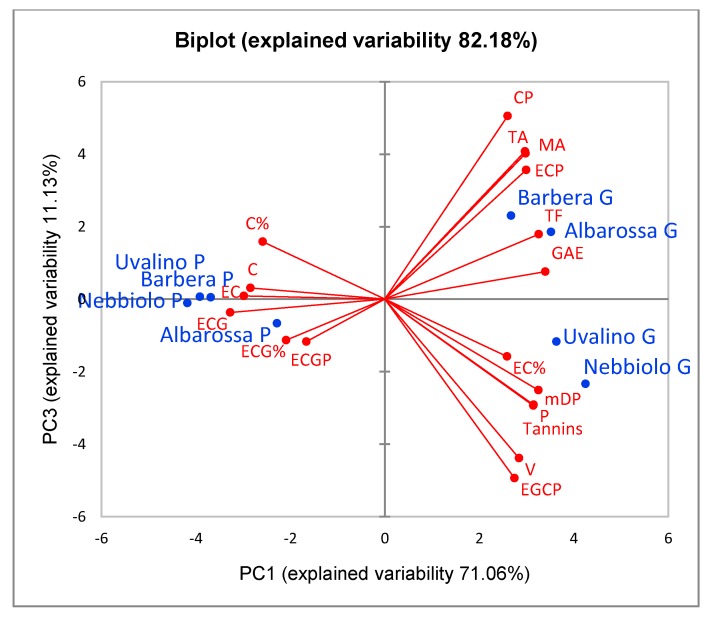
Representation of the loadings (variables) and the scores (skin extracts) in the plane defined by the 1st and 3rd Principal Components (PCA). For each cultivar (blue), the suffix G represents grape extracts, P represents pomace extracts. In red are reported the chemical variables that describe the polyphenolic profile of the extracts: GAE = total polyphenols; TF = total flavonoids; TA = total anthocyanins; MA = monomer anthocyanins; V = flavans reactive with vanillin; P = proanthocyanidins; Tannins = condensed tannins (phloroglucinolysis); mDP = mean degree of polymerization; C, EC, ECG = (+)-catechin, (−)-epicatechin and (−)-epicatechin-3-*O*-gallate present as terminal units; CP, ECP, ECGP = (+)-catechin, (−)-epicatechin and (−)-epicatechin-3-*O*-gallate present as extension units; EGCP = (−)-epigallocatechin present only as extension unit; C%, EC%, ECG% = total percentages of the monomers (terminal + extension).

Figure 1 and Figure 2 show the distribution of the skin extracts from fresh grapes and pomace in the space respectively defined by the 1st and 2nd and the 1st and 3rd Principal Components (PCA). 

The 1st Principal Component, which describes the 71.06% of the total data variability, distinguishes the fresh grape skins from the pomace skins (effect of fermentative maceration on the polyphenolic composition of the skins). It is positively associated with the total polyphenol content (GAE, total flavonoids, total and monomer anthocyanins, vanillin-reactive flavans, proanthocyanidins, condensed tannins), the mean degree of polymerization (mDP) of condensed tannins, and the percentage weights of (+)-catechin, (−)-epicatechin and (−)-epicatechin-3-*O*-gallate present as extension units of condensed tannins. Furthermore, the 1st Component is negatively associated with the percentage weights of the monomers present as terminal units of the condensed tannins. Pomace skins are distinguished from fresh grape skins by the lower content of anthocyanins and tannins, the lower mDP, the higher percentage of monomeric units in terminal position and the higher total percentage content of (+)-catechin. Maceration caused a decrease in the anthocyanins and tannins content of the skins and, in parallel, some modifications of the condensed tannins composition (decrease of mDP and changes in the percentage weight of some monomers that make up the chains).

The 2nd and 3rd Principal Components respectively describe the 15.0% and 11.1% of the total data variability. The 2nd Component mainly distinguishes the Albarossa pomace skins from that of the other cultivars by the highest percentage concentration of (+)-catechin and (−)-epicatechin-3-*O*-gallate in condensed tannins. These differences could be due to different maceration conditions, in particular the duration.

The 3rd Principal Component distinguishes the different cultivars from one another. The distinction mainly concerns the fresh grape skins, while pomace skins are similar to each other. Barbera and Albarossa grapes are distinguished from Nebbiolo and Uvalino grapes, in particular for the total and monomer anthocyanins content, higher in Barbera and Albarossa, and for the content of flavans reactive with vanillin, proanthocyanidins, and condensed tannins, higher in Nebbiolo and Uvalino. The total polyphenol content (GAE), depending on both the anthocyanins and tannins contents, is similar for all cultivars. Moreover, among the extension units of condensed tannins, EGC is more abundant in Uvalino and Nebbiolo, while (+)-catechin is more abundant in Barbera and Albarossa.

**Figure 3:**
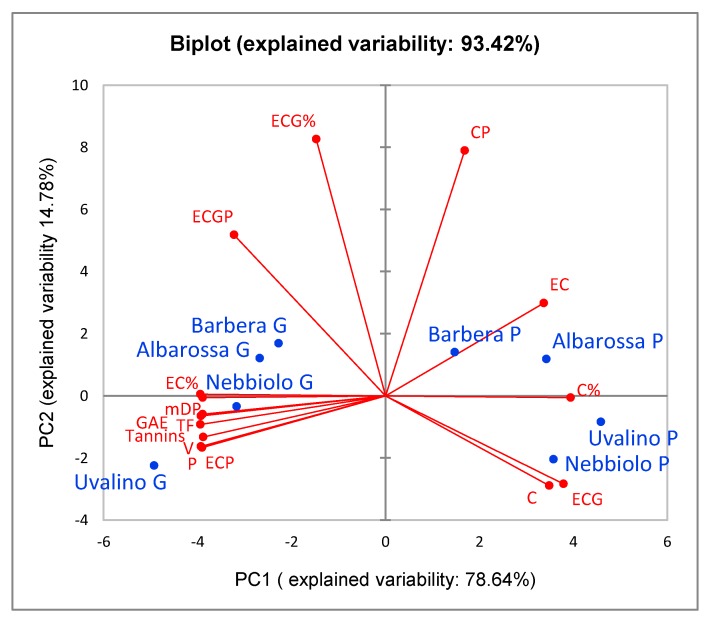
Representation of the loadings (variables) and the scores (seed extracts) in the plane defined by the 1st and 2nd Principal Components (PCA). For each cultivar (blue), the suffix G represents grape extracts, P represents pomace extracts. In red are reported the chemical variables that describe the polyphenolic profile of the extracts: GAE = total polyphenols; TF = total flavonoids; TA = total anthocyanins; MA = monomer anthocyanins; V = flavans reactive with vanillin; P = proanthocyanidins; Tannins = condensed tannins (phloroglucinolysis); mDP = mean degree of polymerization; C, EC, ECG = (+)-catechin, (−)-epicatechin and (−)-epicatechin-3-*O*-gallate present as terminal units; CP, ECP, ECGP = (+)-catechin, (−)-epicatechin and (−)-epicatechin-3-*O*-gallate present as extension units; C%, EC%, ECG% = total percentages of the monomers (terminal + extension).

Considering the seeds, the PCA identified only two Principal Components (93.42% of the total data variability); Figure 3 shows the distribution of seed extracts in the space defined by the two Principal Components. The 1st Principal Component, which describes the 78.64% of the total data variability, distinguishes the grape seeds from the pomace seeds (effect of fermentative maceration on the polyphenolic composition of the seeds). Maceration caused a marked decrease in polyphenolic content of the seeds, and some changes in the condensed tannins composition. In fresh grape seeds the condensed tannins have a higher mean degree of polymerization (mDP) than in pomace seeds, and a consequent lower percentage of monomers in terminal position (C, EC, and ECG). After fermentative maceration, the percentage of monomers as extension units decreased, in particular EC, while the total percentage of C increased.

The second Component (14.78% of the total data variability) distinguishes Barbera and Albarossa from Nebbiolo and Uvalino, in particular for the EGC content, higher in the former than in the latter for both grape seeds and pomace seeds. On the contrary, the differences between cultivars due to the polyphenolic content of the seeds varied after fermentative maceration, and the ranking order of the cultivars for the analyzed parameters changed: Nebbiolo and Uvalino seeds, with the highest tannins content as fresh grapes, show after maceration significantly lower tannins concentrations than Barbera seeds, initially much poorer in tannins.

## 5. Conclusions

During the work, the characterization of the polyphenolic profile of skins and seeds of Albarossa, Barbera, Nebbiolo, and Uvalino, four cultivars extensively cultivated in northern Italy (Piedmont), was carried out. In particular, Barbera and Albarossa grapes had a similar polyphenolic composition and were significantly distinguished from Nebbiolo and Uvalino grapes for many of the analyzed parameters. In particular, regarding grape skins Barbera and Albarossa had a higher content of total and monomer anthocyanins and a lower content of flavans reactive with vanillin and condensed tannins; regarding grape seeds, Barbera and Albarossa had a lower content in flavans reactive with vanillin, proanthocyanidins, and condensed tannins than Nebbiolo and Uvalino. As regards the composition of condensed tannins (phloroglucinolysis results), a lower mDP and a lower percentage of EGC were observed in Barbera and Albarossa skins than in Nebbiolo and Uvalino skins.

The differences in polyphenolic profile observed between the fresh grapes of the different cultivars disappeared after fermentative maceration for almost all the analyzed parameters: the respective pomaces had a composition much more similar to one another. In addition to the duration of the contact between the solid parts of grapes and the fermenting must, the higher homogeneity of polyphenolic composition between the pomaces of different grape cultivars is also the consequence of the management of the fermentative maceration performed by the various wineries: for example, the modalities of the pomace mixing operations (intensity and duration), the adsorbent effect of the yeast strain, the use of maceration enzymes, the temperature, the amount of oxygen supplied, and so on. All these factors have been deeply studied with reference to the polyphenolic composition and color of the wines produced [63], and only sporadically to the composition of exhausted pomace [64]. Unlike the skins, the polyphenolic content of the seeds was extremely reduced and of little interest for the extraction of the polyphenolic fraction. On the contrary, the seeds separated from the grapes before fermentation or during the initial phases of the maceration process could represent an important source of polyphenolic compounds, and at this level the effect of the cultivar on the extraction yields would also be relevant.

As regards the evaluation of the polyphenolic composition of skin and seed extracts, the use of the total polyphenolic index (GAE) alone could be insufficient to discriminate different cultivars from one another: for a better knowledge of the raw material used, in view of the exploitation of byproducts for multiple purposes, it would be advisable to additionally analyze at least the total anthocyanin content by spectrophotometry and the flavanic fraction by spectrophotometry or HPLC. Furthermore, in the case of the skin extracts, the antiradical activity determined with the ABTS method resulted highly and positively correlated only with the flavans content, particularly when determined by phloroglucinolysis and not with the GAE parameter.

For red berry cultivars, the interest in the use of the winemaking byproducts mainly concerns the pomace collected at the end of fermentative maceration (with the sole exception of the pressing pomace derived from the production of white or rosé sparkling wines). Therefore, the prosecution of the work will concern the polyphenolic characterization of a high number of pomace samples of different varieties and provenience, in order to further deepen the knowledge of the potential and the compositional heterogeneity of this raw material, aimed at its industrial exploitation. Conversely, regarding the seeds, a study is in progress on the polyphenolic composition and the antioxidant properties of samples with different varietal origin, sampled during the first days of maceration (*délestage* technique, [63]), when the alcohol content is still low and the losses of polyphenolic compounds are limited.

## Figures and Tables

**Table 1:** Short description of the main extraction methods of polyphenols reported in the literature.

	Reference	Sample Matrix	Sample Treatment	Solvent
**Fresh Grapes**	[27]	Skins and seeds	the intact tissues were extracted separately in covered Erlenmeyer flasks under nitrogen	acetone/water (2:1)
	[28]	Skins	frozen in liquid nitrogen and ground to a fine powder with a grinder	acetone, ethanol various % in water
	[29]	Full berries	triturated with a conventional beater	ethanol/water (1:1), acidified at pH = 2
	[30]	Skins	homogenized in the presence of solvent, then centrifuged	methanol/formic acid (97:3)
	[31]	Skins and seeds	skins: freeze-dried, mill-powdered, freezer; seeds: air-dried, mill-powdered, room temperature	methanol/water/HCl 1N (90:9.5:0.5)
	[17]	Skins and seeds	freeze-dried, ground to a powder using liquid N_2_	methanol at various % in water, then extracts are joined
	[32]	Seeds	finely ground using an ultra-centrifugal mill, then immediately extracted	methanol/water (80:20) followed by acetone/water (75:25)
	[33]	Grape seeds	frozen in liquid nitrogen, then ground with a pestle and a mortar in the presence of solvent	ethyl acetate, then methanol with 5% perchloric acid
**Winemaking Byproducts**	[34]	Distilled pomace	air-dried at room temperature for 48 h, then crushed in a coffee grinder	continuous extraction with either ethanol 100% or water
	[35]	Seeds	sun dried and milled in coffee grinder	ethanol/water (1:1)
	[36]	Pomace	crushed and uncrushed, with and without stems	methanol, ethyl acetate and 3% aqueous KOH
	[37]	Pomace	dried at the ambient temperature and ground	water
	[38]	Pressed marcs	oven dried at 60 °C and milled	ethanol containing different volumes of water (10–20–30–40–50–60%)
	[39]	Marc	dried at 50 °C, then crushed and homogenized with the solvent	ethanol/water (80:20), acidified
	[40]	Pressed marcs	lyophilization and powdering in liquid nitrogen	methanol/water/acetic acid (80:20:5)
	[20,41]	White and red pomace	chopped into small pieces, ground with a pestle and a mortar, and solvent; the paste is again extracted with the same solvent	0.1% HCl in methanol/acetone/water (60:30:10), water/ethanol mixtures and hydrochloric, acetic, or tartaric acid

**Table 2:** Phenolic composition (by spectrophotometry) of the skin and seed extracts expressed as mg/g dry weight (DW) and mg/kg grapes. Antioxidant power ((2,2’-azinobis-(3- ethylbenzothiazoline-6-sulfonate)) (ABTS) method) of the grape skin and seed extracts, expressed as ascorbic acid equivalent in mg/g DW. Standard deviations and ANOVA results.

			Albarossa	Barbera	Nebbiolo	Uvalino	Sig ^2^
**Skins** (Grapes)	mg/g DW	Total anthocyanins	21.5 ± 1.0 d^1^	17.1 ± 1.0 c	9.4 ± 0.8 a	12.4 ± 1.6 b	***
		λ_max_ total anthocyanins (nm)	540.8 ± 0.3 c	540.5 ± 0.0 c	534.2 ± 0.3 a	537.3 ± 0.3 b	***
		Monomer anthocyanins	13.8 ± 1.9 c	11.4 ± 1.3 bc	6.2 ± 0.5 a	7.8 ± 1.5 ab	***
		λ_max_ monomer anthocyanins (nm)	539.7 ± 0.3 c	539.7 ± 0.3 c	533.2 ± 0.3 a	536.5 ± 0.0 b	***
		Monomer/Total anthocyanins	0.64 ± 0.06	0.66 ± 0.04	0.66 ± 0.01	0.63 ± 0.04	ns
		Total flavonoids	47.5 ± 1.3 b	31.2 ± 1.5 a	32.5 ± 1.3 a	33.2 ± 3.3 a	***
		Flavans react. with vanillin (V)	7.2 ± 0.4 a	4.3 ± 0.5 a	20.1 ± 2.4 c	13.5 ± 1.9 b	***
		Proanthocyanidins (P)	24.1 ± 1.9 ab	16.6 ± 1.5 a	43.2 ± 4.6 c	30.5 ± 3.7 b	***
		V/P	0.30 ± 0.04 a	0.26 ± 0.02 a	0.47 ± 0.04 b	0.44 ± 0.03 b	***
		Total polyphenols as gallic acid equivalents (GAE)	37.5 ± 0.6	33.2 ± 0.8	36.7 ± 1.7	34.5 ± 2.9	ns
		ABTS (as ascorbic acid equivalent)	43.5 ± 2.4 ab	34.2 ± 1.5 a	49.7 ± 4.4 b	51.7 ± 4.9 b	***
	mg/kg grapes	Total anthocyanins	2295 ± 109 c	1431 ± 84 b	578 ± 50 a	1220 ± 159 b	***
		Monomer anthocyanins	1475 ± 203 c	948 ± 107 b	382 ± 32 a	769 ± 143 b	***
		Total flavonoids	5073 ± 137 d	2601 ± 123 b	2005 ± 81 a	3262 ± 322 c	***
		Flavans react. with vanillin (V)	773 ± 47 b	359 ± 43 a	1240 ± 149 c	1327 ± 188 c	***
		Proanthocyanidins (P)	2569 ± 205 b	1382 ± 121 a	2660 ± 285 b	2995 ± 364 b	***
		Total polyphenols as GAE	4003 ± 64 d	2771 ± 63 b	2260 ± 103 a	3393 ± 287 c	***
**Seeds** (Grapes)	mg/g DW	Total flavonoids	105.6 ± 0.7 a^1^	128.6 ± 16.1 ab	162.8 ± 21.4 b	158.4 ± 0.1 ab	*
		Flavans react. with vanillin (V)	67.7 ± 1.3 a	75.3 ± 6.1 a	110.4 ± 13.2 b	116.2 ± 0.8 b	***
		Proanthocyanidins (P)	87.4 ± 0.8 a	85.2 ± 3.3 a	125.4 ± 10.9 b	152.0 ± 2.5 c	***
		V/P	0.77 ± 0.02 ab	0.88 ± 0.04 c	0.88 ± 0.03 bc	0.76 ± 0.01 a	*
		Total polyphenols as GAE	73.7 ± 0.2 a	83.8 ± 7.6 ab	106.5 ± 9.0 b	107.8 ± 0.2 b	**
		ABTS (as ascorbic acid equivalent)	109.7 ± 6.8 a	117.6 ± 10.5 a	184.9 ± 7.9 b	185.5 ± 1.3 b	***
	mg/kg grapes	Total flavonoids	5403 ± 34 ab	3962 ± 496 a	8396 ± 1102 c	6933 ± 4 bc	***
		Flavans react. with vanillin (V)	3463 ± 69 a	2318 ± 188 a	5692 ± 680 b	5084 ± 36 b	***
		Proanthocyanidins (P)	4470 ± 40 b	2626 ± 103 a	6467 ± 562 c	6651 ± 111 c	***
		Total polyphenols as GAE	3771 ± 8 b	2581 ± 234 a	5492 ± 465 c	4718 ± 7 bc	***

^1^ Different letters along the line discriminate the trials significantly different from one another (*p* < 0.05, Tukey’s test). ^2^ Significance (Sig): *, **, ***, and ns represent significance at *p* ≤ 0.05, 0.01, 0.001, and not significant, respectively.

**Table 3:** Hydroxycinnamiltartaric acids, flavonols content, and anthocyanins profile of the grape skin extracts. Content of (+)-catechin and (−)-epicatechin in the seed extracts. Standard deviations and ANOVA results.

			Albarossa	Barbera	Nebbiolo	Uvalino	Sig ^2^
**Skins** (Grapes)	HCTA (mg/kg grapes)	cis caffeyl tartatic acid	4.93 ± 0.01 c^1^	3.67 ± 0.00 b	2.78 ± 0.04 a	4.45 ± 0.04 c	***
		trans-caffeyl tartaric acid	8.37 ± 0.27 b	12.64 ± 1.06 c	3.13 ± 0.11 a	14.59 ± 1.57 c	***
		cis p-coumaroyl tartaric acid	1.71 ± 0.06 b	1.45 ± 0.06 b	0.83 ± 0.04 a	1.65 ± 0.26 b	***
		trans p-coumaroyl tartaric acid	1.33 ± 0.06 ab	2.55 ± 0.58 bc	0.12 ± 0.00 a	4.33 ± 1.06 c	***
		trans+cis fertaric acid	1.16 ± 0.24 b	0.91 ± 0.17 b	0.14 ± 0.01 a	1.26 0.11 b	***
	flavonols (mg/kg grapes)	Myricetin	24.8 ± 2.7 ab	64.2 ± 4.3 c	20.5 ± 2.2 a	31.6 ± 2.8 b	***
		Quercetin glucuronide	11.8 ± 1.5 ab	16.6 ± 2.0 c	9.6 ± 0.2 a	14.5 ± 1.7 bc	***
		Quercetin glucoside	17.5 ± 0.3 b	14.6 ± 1.9 b	5.8 ± 0.2 a	14.3 ± 1.9 b	***
		Kaempferol gluc + glucur	7.9 ± 1.1 a	13.8 ± 1.6 ab	10.4 ± 1.5 ab	16.6 ± 4.3 b	*
	monomer anthocyanins profile (% values)	Delphinidin-3-G	17.2 ± 0.7 d	11.2 ± 0.6 c	4.1 ± 0.1 a	6.9 ± 1.1 b	***
		Cyanidin-3-G	7.4 ± 0.1 c	5.3 ± 0.4 b	9.3 ± 0.4 d	2.3 ± 0.2 a	***
		Petunidin-3-G	14.8 ± 0.2 d	13.0 ± 0.5 c	4.1 ± 0.1 a	6.8 ± 0.8 b	***
		Peonidin-3-G	4.9 ± 0.1 a	6.9 ± 0.5 b	48.7 ± 0.1 d	29.1 ± 1.1 c	***
		Malvidin-3-G	34.0 ± 0.2 b	42.5 ± 1.0 d	23.7 ± 0.7 a	38.5 ± 0.3 c	***
		Total acetates	8.0 ± 0.1 b	12.9 ± 0.6 c	3.7 ± 0.1 a	3.1 ± 0.2 a	***
		Total cynnamates	13.6 ± 0.4 c	8.2 ± 0.1 b	6.4 ± 0.3 a	13.2 ± 0.6 c	***
**Seeds** (grapes)	monomer flavan-3-ols (mg/kg grapes)	(+)-Catechin	170 ± 19 b	168 ± 11 b	94 ± 11 a	130 ± 9 ab	**
		(−)-Epicatechin	247 ± 7 a	473 ± 34 b	349 ± 37 a	303 ± 3 a	***

^1^ Different letters along the line discriminate the trials significantly different from one another (*p* < 0.05, Tukey’s test). ^2^ Significance: *, ** and *** represent significance at *p* ≤ 0.05, 0.01 and 0.001, respectively.

**Table 4:** Concentration, monomer percentage composition and mean degree of polymerization of condensed tannins in the grape skins and seeds, and ANOVA results. C = (+)-catechin, EC = (−)-epicatechin, ECG = (−)-epicatechin-3-*O*-gallate, EGC = (−)-epigallocatechin.

			Albarossa	Barbera	Nebbiolo	Uvalino	Sig ^2^
**Skins** (Grapes)	Terminal units (%)	C	5.50 ± 0.40 b^1^	4.73 ± 0.24 b	3.47 ± 0.20 a	3.57 ± 0.32 a	***
		EC	1.42 ± 0.21 c	1.86 ± 0.15 d	0.62 ± 0.05 a	0.98 ± 0.06 b	***
		ECG	0.34 ± 0.07 c	0.18 ± 0.02 b	0.04 ± 0.00 a	0.09 ± 0.01 ab	***
	Extension units (%)	C	20.13 ± 2.17 ab	22.97 ± 0.96 b	17.08 ± 0.49 a	18.01 ± 0.15 a	***
		EC	58.06 ± 2.96 b	58.83 ± 1.03 b	48.15 ± 0.90 a	47.87 ± 0.63 a	***
		ECG	4.35 ± 0.20 c	3.85 ± 0.08 b	2.83 ± 0.09 a	3.86 ± 0.17 b	***
		EGC	10.20 ± 0.59 b	7.58 ± 0.25 a	27.79 ± 1.08 d	25.61 ± 0.63 c	***
	Mean degree of polymerization (mDP)		13.8 ± 0.4 a	14.8 ± 0.8 a	24.2 ± 1.3 c	21.5 ± 1.3 b	***
	Total	C%	25.6 ± 2.4 b	27.7 ± 1.0 b	20.6 ± 0.5 a	21.6 ± 0.2 a	***
		EC%	74.4 ± 2.4 a	72.3 ± 1.0 a	79.4 ± 0.5 b	78.4 ± 0.2 b	***
	Condensed tannins (mg/g DW)		11.3 ± 0.9 b	7.1 ± 0.9 a	19.2 ± 1.0 d	16.0 ± 0.7 c	***
	Condensed tannins (mg/kg grapes)		1202 ± 91 b	594 ± 71 a	1184 ± 64 b	1572 ± 67 c	***
**Seeds** (grapes)	Terminal units (%)	C	6.08 ± 0.22 a^1^	9.70 ± 0.17 c	10.55 ± 0.33 c	7.34 ± 0.23 b	***
		EC	8.10 ± 0.05 b	10.60 ± 0.10 d	8.89 ± 0.10 c	5.74 ± 0.20 a	***
		ECG	5.01 ± 0.14 b	4.37 ± 0.02 a	4.98 ± 0.12 b	4.67 ± 0.20 ab	*
	Extension units (%)	C	14.74 ± 0.51 b	14.75 ± 0.38 b	11.96 ± 1.36 b	5.62 ± 0.64 a	***
		EC	51.56 ± 0.87 b	44.93 ± 0.01 a	50.40 ± 0.97 b	64.10 ± 0.16 c	***
		ECG	14.51 ± 0.06 c	15.64 ± 0.08 d	13.21 ± 0.16 b	12.53 ± 0.17 a	***
	mDP		5.2 ± 0.1 b	4.1 ± 0.0 a	4.1 ± 0.1 a	5.6 ± 0.2 b	***
	Total	C %	20.8 ± 0.7 b	24.4 ± 0.2 c	22.5 ± 1.0 bc	13.0 ± 0.4 a	***
		EC %	79.2 0.7 b	75.5 ± 0.2 a	77.5 ± 1.0 ab	87.0 ± 0.4 c	***
	Condensed tannins (mg/g DW)		53.0 ± 1.4 a	58.4 ± 1.8 a	73.9 ± 5.6 ab	82.5 ± 8.9 b	*
	Condensed tannins (mg/kg grapes)		2712 ± 71 ab	1797 ± 55 a	3810 ± 290 c	3612 ± 390 bc	***

^1^ Different letters along the line discriminate the trials significantly different from one another (*p* < 0.05, Tukey’s test). ^2^ Significance: * and *** represent significance at *p* ≤ 0.05 and 0.001, respectively.

**Table 5:** Phenolic composition in mg/g (DW) of the skin and seed extracts obtained from the fermented pomace of the four studied cultivars, and ANOVA results.

		Albarossa	Barbera	Nebbiolo	Uvalino	Sig ^2^
**Skins** (pomace) mg/g DW	Total anthocyanins	3.68 ± 0.24 c^1^	1.14 ± 0.16 b	0.19 ± 0.02 a	0.44 ± 0.04 a	***
	λ_max_ tot. ant. (nm)	541.0 ± 0.1 c	539.0 ± 0.1 c	533.0 ± 0.1 a	536.0 ± 0.0 b	***
	Monomer anthocyanins	2.94 ± 0.47 c	0.86 ± 0.18 b	0.10 ± 0.01 a	0.26 ± 0.02 ab	***
	λ_max_ mon. ant. (nm)	540.0 ± 0.1 c	538.0 ± 0.2 b	534.0 ± 0.0 a	535.0 ± 0.1 a	***
	Mon/Tot anthocyanins	0.79 ± 0.09 b	0.75 ± 0.06 b	0.53 ± 0.03 a	0.58 ± 0.03 a	***
	Total flavonoids	11.96 ± 1.18 c	6.28 ± 0.58 b	2.55 ± 0.25 a	3.51 ± 0.21 a	***
	Flavans react. with vanillin (V)	0.16 ± 0.07 a	0.82 ± 0.07 c	0.54 ± 0.17 bc	0.48 ± 0.11 b	***
	Proanthocyanidins (P)	3.36 ± 0.41 b	3.31 ± 0.36 b	2.36 ± 0.30 a	1.99 ± 0.08 a	***
	V/P	0.05 ± 0.02 a	0.25 ± 0.02 b	0.23 ± 0.05b	0.24 ± 0.06 b	***
	Total polyphenols as GAE	7.64 ± 0.43 c	6.35 ± 0.53 b	3.45 ± 0.23 a	4.37 ± 0.12 a	***
**Seeds** (Pomace) mg/g DW	Total flavonoids	6.90 ± 0.34 a	25.91 ± 1.21 b	9.51 ± 0.45 a	8.61 ± 0.28 a	***
	Flavans react. with vanillin (V)	0.50 ± 0.02 a	9.35 ± 0.76 b	1.26 ± 0.24 a	1.50 ± 0.34 a	***
	Proanthocyanidins (P)	1.10 ± 0.05 a	13.72 ± 1.06 b	2.81 ± 0.20 a	2.75 ± 0.64 a	***
	V/P	0.44 ± 0.00	0.68 ± 0.00	0.43 ± 0.05	0.57 ± 0.26	ns
	Total polyphenols as GAE	7.10 ± 0.26 a	23.75 ± 1.48 b	9.85 ± 0.42 a	9.52 ± 0.27 a	***

^1^ Different letters along the line discriminate the trials significantly different from one another (*p* < 0.05, Tukey’s test). ^2^ Significance: *** and ns represent significance at *p* ≤ 0.001 and not significant, respectively.

**Table 6:** Concentration, monomer percentage composition and mean degree of polymerization of condensed tannins in the pomace skins, and ANOVA results. C = (+)-catechin, EC = (−)-epicatechin, ECG = (−)-epicatechin-3-*O*-gallate, EGC = (−)-epigallocatechin.

			Albarossa	Barbera	Nebbiolo	Uvalino	Sig ^2^
**Skins** (Pomace)	Terminal units (%)	C	4.72 ± 0.16 a^1^	23.50 ± 0.31 b	35.44 ± 1.34 c	37.97 ± 0.84 d	***
		EC	6.35 ± 0.34 b	9.20 ± 0.17 c	5.22 ± 0.15 ab	4.43 ± 0.82 a	***
		ECG	2.16 ± 0.12 a	3.22 ± 0.25 b	3.41 ± 0.08 b	2.05 ± 0.09 a	***
	Extension units (%)	C	14.05 ± 0.68 a	15.77 ± 0.56 b	14.00 ± 0.36 a	14.03 ± 0.85 a	*
		EC	42.76 ± 0.41 c	35.60 ± 0.64 b	31.99 ± 0.74 a	32.07 ± 1.12 a	***
		ECG	24.91 ± 0.70 c	10.35 ± 0.10 b	6.26 ± 0.17 a	6.38 ± 0.26 a	***
		EGC	5.05 ± 0.07 d	2.33 ± 0.09 a	3.67 ± 0.10 c	3.07 ± 0.12 b	***
	Total	C %	18.77 ± 0.59 a	39.28 ± 0.61 b	49.45 ± 0.99 c	52.00 ± 1.10 d	***
		EC %	81.23 ± 0.59 d	60.72 ± 0.61 c	50.55 ± 0.99 b	48.00 ± 1.10 a	***
	mDP		7.56 ± 0.24 c	2.78 ± 0.05 b	2.27 ± 0.07 a	2.25 ± 0.07 a	***
	Condensed tannins (mg/g DW)		0.85 ± 0.05 a	1.35 ± 0.01 c	1.02 ± 0.08 b	0.95 ± 0.05 ab	***
**Seeds** (Pomace)	Terminal units (%)	C	22.45 ± 0.77 b	16.07 ± 0.16 a	29.39 ± 1.17 c	31.74 ± 0.38 c	***
		EC	18.67 ± 0.41 c	15.36 ± 0.37 b	14.55 ± 0.08 b	12.13 ± 0.27 a	***
		ECG	8.78 ± 0.15 b	5.29 ± 0.08 a	9.15 ± 0.08 b	12.49 ± 0.16 c	***
	Extension units (%)	C	15.18 ± 0.67 b	15.36 ± 0.38 b	9.40 ± 0.32 a	14.98 ± 0.33 b	***
		EC	24.95 ± 0.18 a	34.83 ± 0.17 c	30.90 ± 0.90 b	23.70 ± 0.54 a	***
		ECG	9.96 ± 0.02 c	13.08 ± 0.23 d	6.60 ± 0.58 b	4.95 ± 0.07 a	***
	Total	C %	37.63 ± 0.09 b	31.43 ± 0.22 a	38.80 ± 1.48 b	46.72 ± 0.05 c	***
		EC %	62.37 ± 0.09 b	68.57 ± 0.22 c	61.20 ± 1.48 b	53.28 ± 0.05 a	***
	mDP		2.00 ± 0.02 b	2.72 ± 0.03 c	1.88 ± 0.04 ab	1.77 ± 0.03 a	***
	Condensed tannins (mg/g DW)		1.31 ± 0.05 a	10.34 ± 0.51 b	2.23 ± 0.09 a	2.35 ± 0.11 a	***

^1^ Different letters along the line discriminate the trials significantly different from one another (*p* < 0.05, Tukey’s test). ^2^ Significance: * and *** represent significance at *p* ≤ 0.05, and 0.001, respectively.

## Supplementary Materials

The following are available online at https://www.mdpi.com/2304-8158/8/9/395/s1: Table S1: Average berry weight, average skin weight per berry before and after oven drying, and average freeze-drying yields of the skins (expressed as freeze-dried mass/fresh mass) with indices of data variability (standard deviation and CV%); Table S2: Correlation matrix between the main variables of the polyphenolic and anthocyanin composition and the ABTS parameter, determined for the grape skin extracts of Albarossa, Barbera, Nebbiolo, and Uvalino cultivars; Table S3: Correlation matrix between the main variables of the polyphenolic composition and the ABTS parameter, determined for the grape seed extracts of Albarossa, Barbera, Nebbiolo, and Uvalino cultivars.
