# Supplementary material for: Polyphenolic Characterization of Grape Skins and Seeds of Four Italian Red Cultivars at Harvest and after Fermentative Maceration
Source: Foods. 2019 Sep 6;8(9):395. doi: 10.3390/foods8090395 (PMC6770923; doi:10.3390/foods8090395)
Supplement: Supplementary file 1 [file foods-08-00395-s001.pdf]

Table S1: Average berry weight, average skin weight per berry before and after oven drying, and average freeze-drying yields of the skins (expressed as freeze-dried mass/fresh mass) with indices of data variability (standard deviation and CV%).

	**Albarossa**	**Barbera**	**Nebbiolo**	**Uvalino**
Average berry weight (g)	1.04	2.88	1.75	1.27
Average skin weight/ berry (g)	0.37	0.74	0.37	0.48
Average oven dry skin weight/ berry (g)	0.11	0.21	0.10	0.11
Freeze-drying yield (mean±s.d.)	0.30±0.06	0.32±0.15	0.29±0.04	0.26±0.07
Freeze-drying yield (CV%)	1.73	4.74	1.17	1.93

Table S2: Correlation matrix between the main variables of the polyphenolic and anthocyanin composition and the ABTS parameter, determined for the grapes skins extracts of Albarossa, Barbera, Nebbiolo and Uvalino cultivars.

	Total anthocyanins	Monomer anthocyanins	Total flavonoids	Flavans react. with vanillin	Proanthocyanidins	Total polyphenols as GAE	mDP	Condensed tannins	ABTS
Total anthocyanins	1								
Monomer anthocyanins	0.978	1							
Total flavonoids	0.747	0.708	1						
Flavans react. with vanillin	-0.825	-0.804	-0.266	1					
Proanthocyanidins	-0.752	-0.746	-0.161	0.974	1				
Total polyphenols as GAE	0.140	0,137	0.644	0.383	0.509	1			
mDP	-0.950	-0.935	-0.542	0.929	0.849	0.034	1		
Condensed tannins	-0.781	-0.782	-0.178	0.962	0.959	0.396	0.894	1	
ABTS	-0.554	-0.577	0.017	0.748	0.742	0.391	0.696	0.866	1

Table S3: Correlation matrix between the main variables of the polyphenolic composition and the ABTS parameter, determined for the grape seeds extracts of Albarossa, Barbera, Nebbiolo and Uvalino cultivars.

	Total flavonoids	Flavans react. with vanillin	Proanthocyanidins	Total polyphenols as GAE	mDP	Condensed tannins	ABTS
Total flavonoids	1						
Flavans react. with vanillin	0.951	1					
Proanthocyanidins	0.827	0.954	1				
Total polyphenols as GAE	0.984	0.988	0.908	1			
mDP	-0.114	0.151	0.428	0.028	1		
Condensed tannins	0.878	0.948	0.938	0.30	0.222	1	
ABTS	0.879	0.949	0.913	0.940	0.123	0.895	1
